# Supplementary material for: The role of vaccination and public awareness in forecasts of Mpox incidence in the United Kingdom
Source: Nat Commun. 2023 Jul 11;14:4100. doi: 10.1038/s41467-023-38816-8 (PMC10336136; doi:10.1038/s41467-023-38816-8)
Supplement: Supplementary file 1 — Supplementary Information [file 41467_2023_38816_MOESM1_ESM.pdf]

Supporting information for "The role of vaccination and public awareness in forecasts of Mpox incidence in the United Kingdom"

Samuel P. C. Brand[1,2*], Massimo Cavallaro[1,2,3], Fergus Cumming[4], Charlie Turner[4], Isaac Florence[4], Paula Blomquist[4], Joe Hilton[1,2], Laura M. Guzman-Rincon[1,2], Thomas House[5], D. James Nokes[1,2] and Matt J. Keeling[1,2,3]

[1]The Zeeman Institute for Systems Biology Infectious Disease Epidemiology Research (SBIDER), Coventry, United Kingdom.
[2]School of Life Sciences, University of Warwick, Coventry, United Kingdom.
[3]Mathematics Institute, University of Warwick, Coventry, United Kingdom.
[4]United Kingdom Health Security Agency, United Kingdom.
[5]Department of Mathematics, University of Manchester, Manchester, United Kingdom.

*Corresponding author(s). E-mail(s): S.Brand@warwick.ac.uk;

Appendix S1 Supporting Information

S1.1 Reproductive number and case distribution for Mpox

The next-generation matrix [1] R_{ij}, that is the expected number of infections in group i caused by a single infected person in group j is,

$$\begin{aligned} R_{ij} &= \frac{N_i}{N} R_{\text{other}}(t) + \frac{\mu_j \mu_{inf} p_{\text{GBMSM}}(t)}{10}, \qquad i,j > 0, \\ R_{0,j} &= \frac{N_0}{N} R_{\text{other}}(t), \qquad j > 0, \\ R_{i,0} &= \frac{N_i}{N} R_{\text{other}}(t), \qquad i > 0, \\ R_{0,0} &= \frac{N_0}{N} R_{\text{other}}(t). \end{aligned} \tag{A1}$$

Where the groups $i = 0, 1, \ldots, 10$ are the non-GBMSM then the 10 GBMSM sexual activity groups, and N_i are the group sizes. The basic reproductive number R_0 can be calculated numerically by evaluating the leading eigenvalue, and the idealized case distribution is the associated eigenvector (normalized to sum to 1). The reproductive number for only sexual partnerships within the GBMSM can be evaluated directly by noting that the idealized case distribution is flat by construction over the sexual activity groups, that is we chose the group sizes so this would be true under a random partnership model (see subsection 4.2 main text),

$$\text{GBMSM } R_0(t) = \mu_{inf} p_{\text{GBMSM}}(t) \frac{1}{10} \sum_{j=1}^{10} \mu_j. \tag{A2}$$

The full R_0 including both sexual partnership transmission among GBMSM and other transmission pathways can be calculated numerically from equation (A1) using an eigenvalue solver. However, because the non-GBMSM population is much larger than the GBMSM population $N_0 \gg N_i$ for $i = 1, \ldots, 10$ we can get a good approximation to the full reproductive number $\tilde{R}_0$ by setting $N_0/N = 1$ and $N_i/N = 0$ for $i = 1, \ldots, 10$. This is equivalent to assuming that all other transmission, apart from sexual partnership among GBMSM, occurs in the non-GBMSM population, which is a reasonable approximation. The approximate $\tilde{R}_0$ has an analytical solution,

$$\tilde{R}_0(t) = \max\{\text{GBMSM } R_0(t), R_{\text{other}}(t)\}. \tag{A3}$$

Assuming that GBMSM $R_0(t) > R_{\text{other}}(t)$ the proportion of non-GBMSM cases is,

$$\frac{C_{\text{other}}(t)}{C_{\text{other}}(t) + C_{\text{GBMSM}}(t)} = \frac{R_{\text{other}}(t)}{\text{GBMSM } R_0(t)} \tag{A4}$$

The same argument as above applies to an instantaneous form of the reproductive number GBMSM $R(t)$, which accounts for population immunity due to infections and vaccinations. Therefore, as noted in the main text, we should expect the proportion of cases among non-GBMSM to increase because the effect of infections and vaccinations in building population immunity is more significant among the GBMSM population compared to the non-GBMSM population which increases the size of the fraction in equation (A4).

S1.2 Prior predictive checking and simulation based calibration

Following a Bayesian workflow [2] we first generated 1000 parameter samples from the prior distribution π: $\theta^{(i)} \sim \pi$ for $i = 1, ..., 1000$. Then for each parameter sample we simulated a trajectory for weekly GBMSM and non-GBMSM cases $\hat{C}^{(i)}_{\text{GBMSM}}(w)$, $\hat{C}^{(i)}_{\text{other}}(w)$, $i = 1, ..., 1000$. These simulations from the prior distribution were visually inspected to assess the coherence of the prior beliefs, as represented by priors on parameters, with our prior belief on the trajectory of MPXV in the United Kingdom. This demonstrated a reasonably wide spread of *a priori* potential outcomes, including early disappearance (Fig. S1). Our *a priori* belief, prior to inference, was that MPXV transmission outside of prolonged physical contact would be low, and our prior predictive checking accords with this (Fig. S1).

Before running the SMC-ABC inference we generated a target error threshold for accepting a parameter into an empirical ensemble approximating the Bayesian posterior distribution using simulation based calibration. The procedure was to resample a trajectory for weekly GBMSM and non-GBMSM cases for each of the prior draws $\theta^{(i)}$: $\tilde{C}^{(i)}_{\text{GBMSM}}(w)$, $\tilde{C}^{(i)}_{\text{other}}(w)$ and generate an ensemble of error measures: $d_1(\hat{C}^{(i)}, \tilde{C}^{(i)})$ for $i = 1, ..., 1000$. This ensemble represented a spread of typical errors between case trajectories even when both trajectories were generated using the same underlying parameters. The target error value for ABC-SMC parameter acceptance, target, was chosen to be at the 25th percentile of the generated error measure ensemble (Fig. S1).

S1.3 Fixed and estimated parameter values

Descriptions and either fixed values/ranges or posterior estimates for parameters used in the transmission model. Fixed or fixed range parameter values are given in table S1. Prior and posterior distribution statistics for inferred parameters are given in both tabular form (table S2), and as a plot (Fig. S2).

S1.4 Inferred metapopulation structure for GBMSM

As described in *Methods* we inferred a posterior distribution for the dispersion parameter (α_m) of the clique size distribution within the GBMSM sexual contact metapopulation $\mathbf{n}_{meta} \sim \text{DirichletMultinomial}(N_{\text{GBMSM}}, \alpha_m \mathbf{1})$. Generating a series of ordered (from largest to smallest size) samples $\mathbf{n}^{(i)}_{meta}$

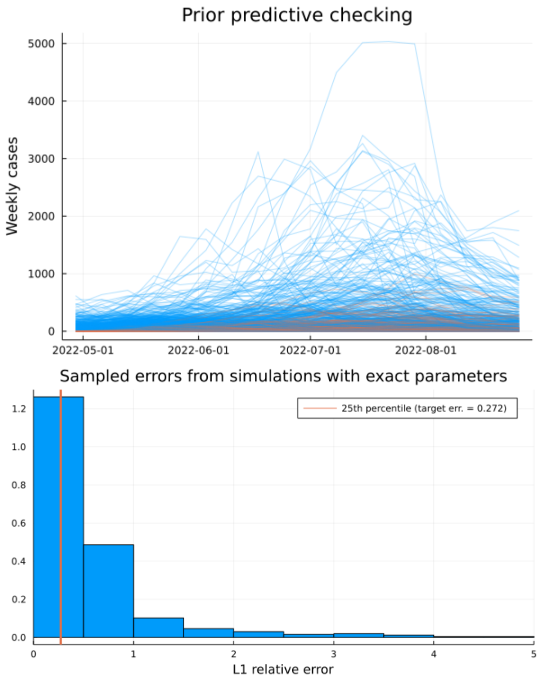

Fig. S1 Prior predictive checking and simulation based calibration. Trajectories of weekly cases for GBMSM (blue lines) and non-GBMSM (red lines) as generated using the prior distribution on parameters (*top*). The distribution of errors when resampling trajectories using matched parameters with the 25th percentile (red line) chosen as the SMC-ABC inference threshold (*bottom*).

for $i = 1, \ldots, 2,000$ gives a posterior prediction for the inferred underlying metapopulation structure for sexual transmission within the GBMSM community. The inference suggests that the GBMSM population is well-connected as opposed to fragmented into smaller subpopulations, since posterior draws from the clique size distribution typically have 10 or fewer groups of significant size with the largest group forming nearly 50% of the GBMSM population within the 95% CI (Fig. S3).

S1.5 Scenario-based Projections of MPox Case Incidence

From SMC-ABC inference (see subsection 4.7 main text) we generated 2000 accepted epidemic trajectories. For each of the $n = 1, \ldots, 2000$ **accepted** trajectories we saved:

- The model parameters $\{\theta^{(n)}\}_{n\geq 1}$. These are used to approximate the posterior distribution of model parameters, $P(\theta \mid C_{\text{GBMSM}}, C_{\text{other}})$.
- The generated case trajectories $\{\hat{C}^{(n)}_{\text{GBMSM}}, \hat{C}^{(n)}_{\text{other}}\}_{n\geq 1}$. We used these trajectories when showing model projections compared to data (Fig. 1 and 2 main text).

Parameter	Fixed value	Description
Mean generation time.	9.25 days	Mean time to secondary infections [3].
Mean incubation time.	7.75 days	Mean time from infection to symptoms [3].
p_{inc}	0.258	Daily probability of latency progression (fitted).
ϵ	0.776	Relative infectiousness of of pre-symptomatic infected (fitted to 50% pre-symptomatic infections with fixed mean generation time).
p_{inf}	0.333	Daily probability of progression from symptomatic infectious (fitted).
Effective infectious period.	6.01 days	Effective infectious duration.
N	67.2 millions	Population size of United Kingdom.
N_{gbmsm}	761.0 thousands	GBMSM population size of United Kingdom [4, 5].
$f(k) \sim k^{-\alpha}$	$\alpha = 1.81$	Power-law distribution for rate of new sexual contacts for GBMSM people [4].
v_{eff}	$\mathcal{U}(0.7, 0.85)$	Reduction in susceptibility of vaccinated people assumed to be lower than fully vaccinated estimate of 85% [6, 7].
T_2	23rd July	Date of WHO announcement that Mpox is a public health emergency of international concern (PHEIC) [8]
T_r	15th Sept or 13th Oct	Mid-point times for reversion to baseline behaviour (scenarios).

Table S1 Definitions of the fixed parameters.

- The full state of the model at four time points:
  1. The initial state of the model, $\{\mathcal{S}^{(n)}_{init}\}_{n\geq 1}$.
  2. The week before first vaccinations, $\{\mathcal{S}^{(n)}_{prevac}\}_{n\geq 1}$.
  3. The week before behaviour reversion, $\{\mathcal{S}^{(n)}_{prerev}\}_{n\geq 1}$.
  4. The final week of data, $\{\mathcal{S}^{(n)}_{end}\}_{n\geq 1}$.

For scenario projection of behaviour reversion, e.g. 4 or 12 week reversion period, we generated 2000 case trajectories such that the nth projection used model parameters $\theta^{(n)}$ with initial state on Monday 5th September 2022 $\mathcal{S}^{(n)}_{prerev}$. For the counter-factual scenarios without vaccines, we generated 2000 case trajectories such that the nth projection used model parameters $\theta^{(n)}$ with initial state on Monday 11th July 2022 $\mathcal{S}^{(n)}_{prevac}$.

S1.6 Model diagnostics and alternative model choices

In this section we assess the model fit to the Mpox case data in the United Kingdom, and consider alternative model formulations.

The transmission model used in this paper is based on survey-based studies finding evidence of awareness of Mpox symptoms with intention to reduce risk

Parameter	Posterior mean (95% CI)	Prior distribution	Description
Population structure and initial condition parameters			
α_m	2.36 (0.284, 5.73)	$Exp(1)$	Dispersion parameter for metapopulation sizes.
ι_0	6.11 (0.861, 14.9)	$LogNormal(\ln(5), 1)$	Scale parameter for the number of initial infected people.
Detection probability parameters			
p_d	0.454 (0.167, 0.869)	$Beta(5, 5)$	Mean weekly probability of case detection. Weekly probability of detection was random with $P_w \sim Beta(\alpha, \beta)$ with $\mathbb{E}[P_w] = p_d$.
$\Phi_d = \frac{1}{M+1}$	0.00156 (0.000317, 0.00592)	$M \sim Gamma(3, 1000/3)$	Dispersion of weekly probability of case detection (M is the effective sample size $\alpha + \beta$ for the weekly $P_w \sim Beta(\alpha, \beta)$ distributed probability of case detection).
Baseline transmission parameters			
$p_{gbmsm}(0)$	0.434 (0.249, 0.777)	$Uniform(0, 1)$	Baseline probability of transmission per sexual contact.
$R_{other}(0)$	0.0398 (0.00898, 0.0716)	$LogNormal(\ln(0.25), 1)$	Baseline reproductive number non-GBMSM sexual contacts.
Behaviour and risk change point parameters			
T_1	11-June (18-May, 13-July)	U(15 May, 18 July)	Change point time for reduction in transmission due to awareness of MPX.
$\rho_{gbmsm,1}$	0.381 (0.0753, 0.655)	$Beta(1.5, 1.5)$	Reduction in GBMSM reproduction number after change point at T_1.
$\rho_{gbmsm,2}$	0.338 (0.0466, 0.633)	$\rho_{gbmsm,2} \sim Beta(1.5, 1.5)$	Further reduction in GBMSM reproduction number after WHO announcement of PHEIC.

Table S2 Definitions of the target parameters for inference and their posterior estimates given as posterior means with 95 % credible intervals.

of either spreading or contracting Mpox becoming widespread in various high-income countries by the second half of 2022 [9–12], but also that awareness of Mpox was higher among GBMSM people compared to the general population [12]. These observations lead us to favour a transmission model which included the possibility of infected GBMSM people reducing their transmission potential via new sexual contacts over time due to behaviour change, whereas the background homogeneous transmission per infected doesn't change with time. Additionally, we assume that there is a random number of metapopulation

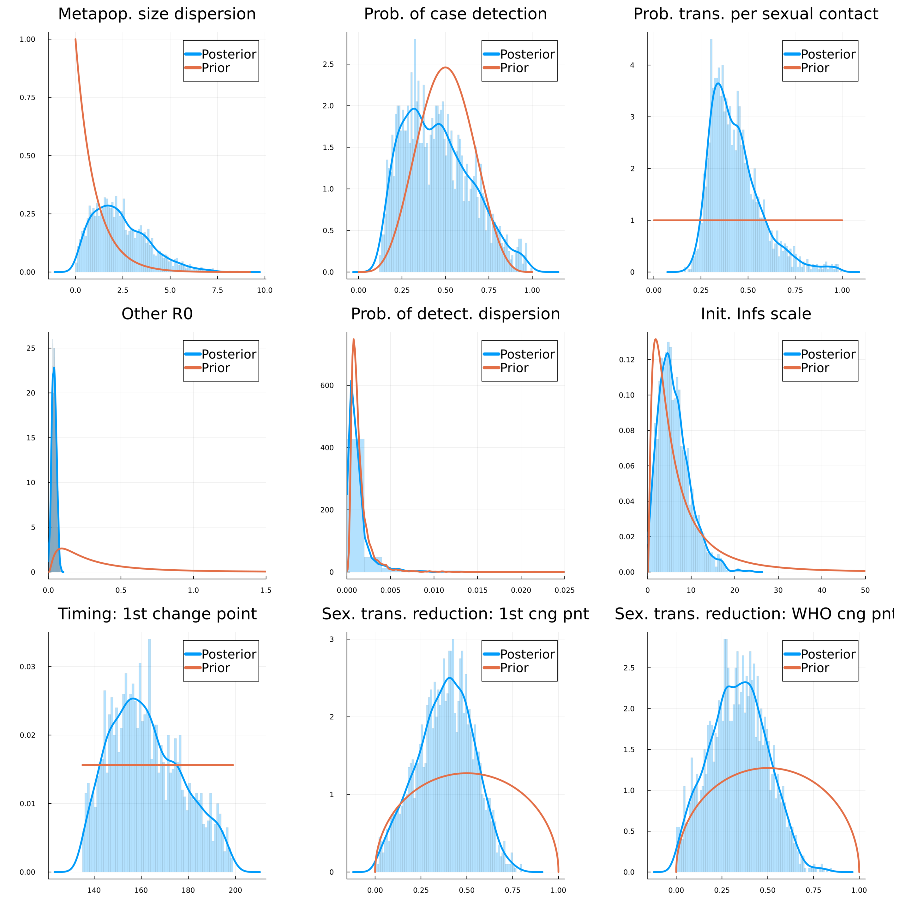

Fig. S2 Empirical posterior distributions of the target parameters for inference. Blue and red colours indicate, posterior and prior distributions, respectively. Posterior draws are shown both as a histogram (*bars*) and with a smooth density approximation (*curves*). Histograms are from $n = 2000$ parameter sets drawn from their posterior distribution.

sub-groups among the GBMSM population as an approximation to individual based model transmission dynamics.

However, we also consider alternative transmission model formulations as a sensitivity analysis. The goal of this sensitivity analysis is, (i) check if other model formulations can be better calibrated to the Mpox case data than the main model used in this paper, and, (ii) to elucidate on the role of different model features in medium-term forecasting of case incidence trends.

S1.6.1 Model checking

We treat the calibrated model simulated case trajectories (see Fig. 1 main text) as approximations to sampling from the posterior distribution of *possible* case trajectories, $\{\tilde{C}^{(k)}_{\text{GBMSM}}, \tilde{C}^{(k)}_{\text{other}}\}_{k\geq 1}$, under our main model choice, conditional

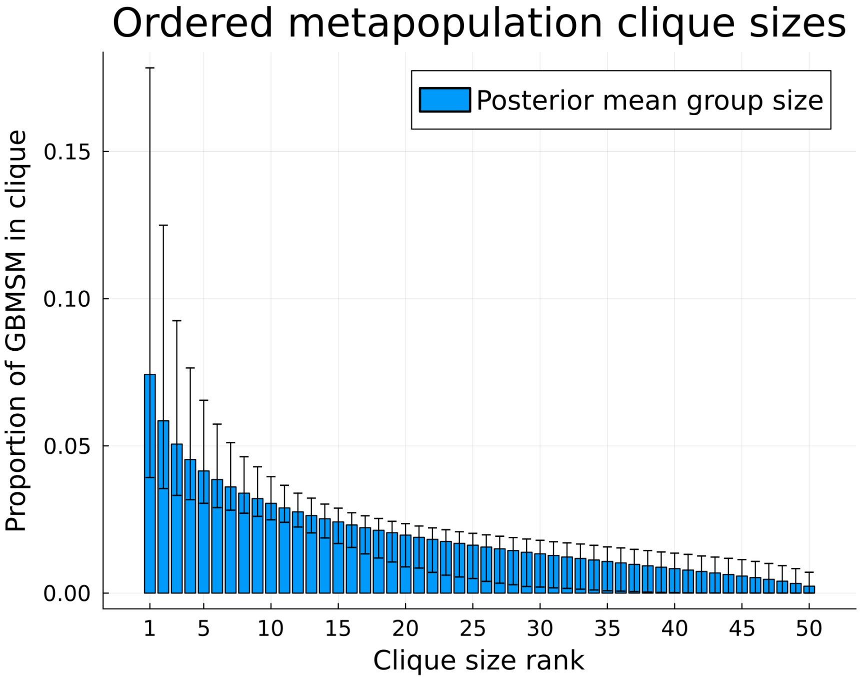

Fig. S3 Distribution of ordered metapopulation sizes. The posterior mean size, relative to the total size of the GBMSM population, of each metapopulation in the overall metapopulation ordered by size is shown as bars, the 95%CI are shown as errors. Metapopulation size distributions are based on $n = 2000$ parameter sets drawn from their posterior distribution.

on the actually observed case data, $\mathbb{P}(\tilde{C}_{\text{GBMSM}}, \tilde{C}_{\text{other}} \mid C_{\text{GBMSM}}, C_{\text{other}})$. From the ensemble of $n_{sample} = 2000$ samples of case trajectories, $\tilde{C}_{\text{GBMSM}}$, $\tilde{C}_{\text{other}}$, we can marginalise to estimate posterior marginal probabilities,

$$F_{\text{GBMSM},w} = \frac{1}{n_{sample}} \sum_{k=1}^{n_{sample}} \tilde{C}^{(k)}_{\text{GBMSM}}(w) \leq C_{\text{GBMSM}}(w), \qquad w = 1, 2, \dots,$$
$$F_{\text{other},w} = \frac{1}{n_{sample}} \sum_{k=1}^{n_{sample}} \tilde{C}^{(k)}_{\text{other}}(w) \leq C_{\text{other}}(w), \qquad w = 1, 2, \dots. \tag{A5}$$

If the predictive distribution $\mathbb{P}(\tilde{C}_{\text{GBMSM}}, \tilde{C}_{\text{other}} \mid C_{\text{GBMSM}}, C_{\text{other}})$ is well calibrated then the ensemble of $F_{\text{GBMSM},w}$, $F_{\text{other},w}$ values should be a probability integral transform (PIT) values for the predictive distribution, and therefore at least approximately uniformly distributed [13, 14]. The main model had PIT values that were reasonably similar to uniform distributed (Fig. S4).

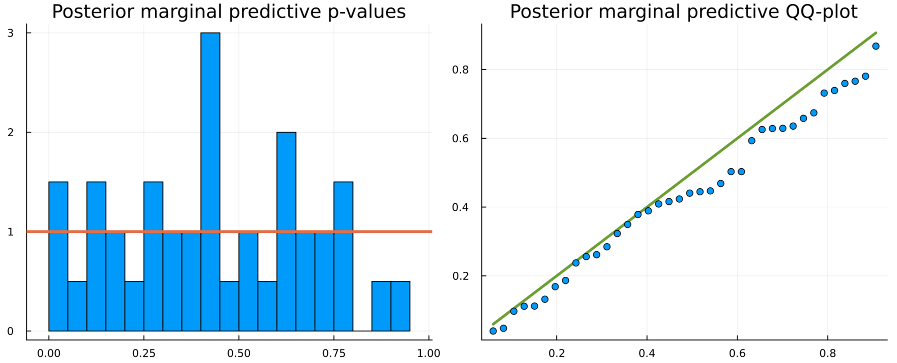

Fig. S4 **Model diagnostic plots**. *Left*: Histogram of PIT values for marginal predictions of weekly cases with target uniform density (*solid line*). *Right*: Quantile-quantile (QQ) plot of PIT values.

S1.6.2 Model-choice sensitivity analysis and sequential projections using redacted data

The alternate model formulations considered here are:

- *Full model.* A model extension to include the possibility of the reproductive number for other transmission pathways R_{other} changing over time,
$$\frac{R_{\text{other}}(t)}{R_{\text{other}}(0)} = \Big[1(t \geq T_2)(1-\rho_{\text{other},2})\Big]\Big(1-\rho_{\text{other},1}\sigma((t-T_1)/\kappa_1)\Big) + \Delta\rho_{\text{other}}\sigma((t-T_r)/\kappa_r). \tag{A6}$$
Where $\rho_{\text{other},1}$ and $\rho_{\text{other},2}$ are the proportional risk reductions of other transmission pathways due to behavioural change and a reaction to the WHO announcement of a public health of emergency of international concern, respectively, and $\Delta\rho_{\text{other}} = 1-(1-\rho_{\text{other},1})(1-\rho_{\text{other},2})$ is the proportionate change from 1st May to the minimum point after T_2. When fitting this model we used uninformative priors, $\rho_{\text{other},1} \sim \text{Beta}(1.5,\ 1.5)$, $\rho_{\text{other},2} \sim \text{Beta}(1.5,\ 1.5)$.
- *No behaviour change.* A model with no behaviour change; in this model all the parameters and their priors are the same as the main model except $\rho_{\text{other},1} = \rho_{\text{other},2} = 0$.
- *One metapopulation.* A model with one metapopulation; in this model all the parameters and their priors are the same as the main model except that all GBMSM individuals are in the same metapopulation group, therefore new sexual contacts between GBMSM individuals form according to the random partnership model.

We refitted using case data only available in the first 4, 8, 12 and 16 weeks and generated 12 week look-ahead projections from the posterior distribution of parameters and final model states for each refit (Fig. S5). That is that the nth look-ahead projection being generated using the nth accepted parameter set $\theta^{(n)}$ with initial state on the last week of redacted data, $\{\mathcal{S}^{(n)}_{end}\}$. As with

the main results, the first two weeks and last week case data were excluded from the distance function. For the week 8, 12... projections we also reran the GBMSM data imputation (see subsection S1.7) using only case data available at the time, for week 4 projections there was insufficient data so we used the frequency of identified GBMSM status cases (96.7%) to make the probabilistic weights for cases without GBMSM status assigned. When forecasting from 4 or 8 weeks of initial data, we are notionally before the WHO announcement of a public health emergency of international concern (PHEIC), therefore, models that used this as an effect on transmission had this effect set to zero. On the other hand, understanding of key epidemiological quantities such as the Mpox mean generation time, and the vaccination rates are treated as fixed for all sequential forecasts. Therefore, these projections are not fully retrospective forecasts, since they use leading information ahead of the data used in inference. Rather, the projections are a sequential comparison between model-based projections with GBMSM status inference and actual data.

We compare the various model formulations by prediction accuracy over a 12 week forecast horizon using the same scoring function as used for the ABC acceptance threshold (equation 10 main text) where there was an overlap between forecast case trends and actual case data. We considered both scoring each model and each refit by $d(\hat{C}_{median}, C)$, where $\hat{C}_{median}$ was the posterior median case projection over the forecast ensemble for the model and refit combination, and by the statistics of the ensemble of errors $\{d(\hat{C}^{(k)}, C)\}_{k\geq 1}$, where $\hat{C}^{(k)}$ was the kth case forecast in the ensemble for the model and refit combination (Fig. S5, Table S3).

Overall, the main model and the full model had similar forecast errors, averaged over different data redaction points. For these two models we found that in each sequential projection before or at the actual peak in case detection, the posterior median for cases among GBMSM and non-GBMSM people peaked in June or July below 550 cases per week, in reasonable agreement with actual data (actual peak was in mid-July with 430 cases that week). For each projection using these models past the peak, the posterior median for cases projected a decline in cases with low case rates by the end of September 2022, in agreement with the actual data (Fig. S5). However, when fitted on the full data set the full model under-estimates the number of non-GBMSM cases in the decline phase of the outbreak (Fig. S6).

The models with less parameters than the main model, having either no behavioural change or only one metapopulation, generally performed worse at forecasting than the main model or the full model (Fig. S5, Table S3), especially when fitted on earlier data. However, by September 2022 all models could retrospectively fit the observed cases well (Fig. S5), and the "no behaviour change" model was the best at forecasting the rate of decline of cases (Table S3).

The "one metapopulation" model assigned higher probability to faster future changes in case rates, either faster increases or faster decreases, compared to the main model (Fig. S5). We interpret this as being due to new

Forecast error: Median projection				
Projection date	Main model	Full model	No behaviour change	one metapopulation
23/05/2022	0.694	0.426	2.590	3.394
20/06/2022	0.557	0.517	2.090	0.686
18/07/2022	0.428	0.452	1.253	0.711
15/08/2022	0.655	0.709	0.371	0.747
Forecast error: Median error (95% PI)				
Projection date	Main model	Full model	No behaviour change	one metapopulation
23/05/2022	1.311 (0.458, 25.039)	0.999 (0.380, 14.328)	4.11 (0.4934, 47.129)	4.326 (0.717, 73.512)
20/06/2022	0.844 (0.367, 3.380)	0.821 (0.306, 3.376)	2.337 (0.471, 7.647)	0.705 (0.435, 0.901)
18/07/2022	0.561 (0.205, 1.318)	0.556 (0.240, 1.178)	1.353 (0.251, 4.782)	0.709 (0.386, 0.908)
15/08/2022	0.658 (0.297, 0.971)	0.714 (0.340, 0.984)	0.540 (0.305, 2.456)	0.737 (0.4236, 0.961)

Table S3 Forecast errors for different models by start date of forecasting. Each model variant was fitted on data before the projection date. *Top block*: Forecast error for the median projection of case incidence. *Bottom Block*: Spread of forecast errors over projections, shown as median and 95% prediction intervals.

sexual contacts between GBMSM people occurring among a well mixed population within the "one metapopulation" model. In contrast, the main and "full" models assign some probability to structure in new sexual contacts among GBMSM people and, therefore, assigns higher probability to relatively slower growth in the early phase and relatively more persistence in the end phase of the epidemic curve. On the other hand, at the projection date with 8 weeks of data (20/06/2022) although the "one metapopulation" model has a less accurate median projection over forecast cases, it has a lower variance prediction compared to the main model and better median error over all predictions (Table S3). The "no behaviour change" model was slowest to react to emerging evidence that the rate of new cases was diminishing, and consistently over estimated the time to peak and peak size until mid-August data was available (Fig. S5).

Overall, all the models eventually find a posterior distribution for parameters that allows each model to have a good fit to the case data (Fig. S5) using the uninformative priors used in this paper. Projecting each model forwards on the complete set of data finds agreement across models that the case rate is likely to drop to zero (Fig. S6). Our reason for preferring the main model, that is that there is an effect on transmission due to behaviour change among GBMSM, is based on two observations: (i) survey studies not used in inference find evidence of behaviour change among GBMSM respondants [9–12], and (ii) the main model outperforms the simpler model formulations in sequential forecasting.

S1.6.3 Uniform Vaccination of GBMSM

In this section we consider the counter-factual that, rather than Mpox vaccines being offered to the most sexually active GBMSM individuals (defined in this

study as those with typically at least one new sexual partner per month), doses of Imvanex/Jynneos were offered to all sexually active GBMSM individuals (including GBMSM individuals with less than one new sexual partner per month). In this counter-factual we have increased the target group encouraged to come forwards for vaccination without increasing the number of available first doses, and, under the assumption that doses are sought proportionally by group size, 91% of doses were received by GBMSM individuals who have less than one new sexual partner per month on average.

In line with results presented in the main manuscript indicating a heavy skew in risk towards the higher sexual activity groups, we find that a uniform vaccination strategy is less effective than opening vaccines only to more sexually active GBMSM people. In fact, the counter-factual projection of weekly case incidence under uniform vaccination of GBMSM is very close to the counter-factual projection of weekly case incidence without vaccines (Fig. S7). The implication from the counter-factual modelling is that encouraging only the most at-risk GBMSM individuals to seek vaccination was an appropriate strategy given limited doses of Imvanex.

S1.7 GBMSM data imputation

Data on Mpox cases were maintained by UKSHA, updated every week date until the 6th of October, and incorporate information from several sources – such as patient response to questionnaires, contact tracing data collected by Health Protection Teams (HPTs), and laboratory reports. A total of 69 variables were included, encoding for dates of symptom onset, laboratory reports, travel dates and destination, incident number from HPTs, ethnicity, gender, sex, sex at birth, and sexual behaviour during the 21 days prior symptoms, whether the patient is a healthcare worker, and locations (upper-tier local authorities and regions) of patients' addresses and hospitals from which laboratory results were sent. A binary variable to indicate whether a patient identifies as GBMSM was also included. Many fields contain missing data, arguably due to patients compiling only parts of the questionnaires.

In particular, we are interested in the imputation of missing GBMSM values. We split data into a learning set, consisting of cases with reported GBMSM status, and an imputation set, which included all the cases with missing GBMSM status. Fields containing more than 500 missing entries were removed and those remaining were used as predictor variables to inform gradient-boosted decision tree (GBDT) classifiers, trained to predict the probability that a case is GBMSM. Often in imputation this probability is translated into a binary variable, but here the probability is sufficient to derive the expected fraction of GBMSM among those who did not answer. GBDT models were particularly useful with this database as they automatically handle missing data and are essentially unaffected by collinearity of predictors. All dates were converted to day numbers and all categorical variables were stratified (one-hot encoding).

Parameter name	20/06/2022	18/07/2022	15/08/2022	17/09/2022	26/10/2022
`subsample`	0.7	0.5	0.5	0.5	0.5
`colsample_bytree`	0.5	0.7	0.4	0.7	0.7
`max_depth`	5	10	10	4	9
`eta`	0.7	0.5	0.3	0.3	0.5
`nrounds`	23	23	18	28	23
`min_child_weight`	0	0	0	0	0
`scale_pos_weight`	0.19	0.20	0.24	0.25	0.26

Table S4 Xgboost hyper-parameters were selected by means of minimisation of RMSE using 2-fold cross-validation over the learning set with constant L1-regularisation parameter $\alpha = 0.5$ for each outcome. Parameter `scale_pos_weight` was computed as the square-rooted ratio of non-GBMSM over GBMSM reported case numbers. Columns corresponds to parameters obtained from data available in the week starting from 20/06/2022, 18/07/2022, 15/08/2022, 12/09/2022, and 26/09/2022.

GBDT models with logistic objective function were implemented in the XGBoost library (v0.81) [15]. These models depend on a set of hyper-parameters which were selected from the learning sets by minimising the average root-mean square error (RMSE) of the predictions obtained by 2-fold cross validation with grid search. We repeated model building and imputation recursively using data available in the weeks starting from 20/06/2022, 18/07/2022, 15/08/2022, 12/09/2022, and 26/09/2022 (included) to illustrate how predictions were updated as new cases are included in the data set. The selected hyper-parameters are reported in Table S4. GBDT models with such hyper-parameters were trained over the learning set and the classifiers with optimal RMSE (0.17, 0.16, 0.15, 0.15, and 0.15 for imputations performed in the weeks starting from from 20/06/2022, 18/07/2022, 15/08/2022, 12/09/2022, and 26/09/2022, respectively) were used to predict GBMSM status over the imputation set. The imputed value for the case i whose GBMSM value is missing consists of the probability P_i that i can be identified as GBMSM. Averaging over all trees the information gain across all splits a feature is used in, it is possible to rank all features by their overall importance for the prediction (regardless of whether the feature contributes a negative or positive term to the prediction, see, e.g., [16, 17]). As illustrated in Table S5, patient sex unsurprisingly is the most important predictor, followed by specimen collection and reporting dates and some locations of the HPTs and hospitals who managed the cases (arguably due to epidemiologically linked cases being collected and reported in spatial and temporal proximity). While it is impossible to predict the GBMSM status of each single case with certainty, the probability we obtained is highly informative after aggregation. The number of cases imputed GBMSM in week w is $G(w) = \sum_i X_i I_i(w)$, where $X_i \sim Bernoulli(P_i)$ and $I_i(w) = 1$ if case i was reported in week w, $I_i(w) = 0$ otherwise. The expectation $E[G(w)]$ is obtained by simulation and gives the expected fraction $p(w) = E[G(w)]/C_{\mathrm{NA}}(w)$ to be used in equation 9 of the main text. Estimates obtained from data are illustrated in Figure S8.

Feature	Information gain
Sex female	4.57×10^{-1}
Specimen collection date	8.64×10^{-2}
Laboratory result report date	8.61×10^{-2}
Date reported to HPZone	7.12×10^{-2}
Date reported to IMT	6.60×10^{-2}
Sex male	2.86×10^{-2}
Hospital location East Midlands	1.89×10^{-2}
HPT South West London	1.86×10^{-2}
Hospital location London	1.43×10^{-2}
Hospital location South East	1.32×10^{-2}
HPT North East	1.20×10^{-2}
Date added to linelist	1.17×10^{-2}
Hospital location East of England	1.13×10^{-2}
Hospital location West Midlands	1.03×10^{-2}
HPT Bedfordshire and Hertfordshire	9.85×10^{-3}

Table S5 Top 15 features included in the GBDT model ranked by their information gain.

References

[1] Diekmann, O., Heesterbeek, J., Roberts, M.G.: The construction of next-generation matrices for compartmental epidemic models. Journal of the royal society interface **7**(47), 873–885 (2010)

[2] Gelman, A., Vehtari, A., Simpson, D., Margossian, C.C., Carpenter, B., Yao, Y., Kennedy, L., Gabry, J., Bürkner, P.-C., Modrák, M.: Bayesian Workflow (2020) arXiv:2011.01808. https://doi.org/10.48550/arxiv.2011.01808

[3] Ward, T., Christie, R., Paton, R.S., Cumming, F., Overton, C.E.: Transmission dynamics of monkeypox in the united kingdom: contact tracing study. bmj **379** (2022)

[4] Whittles, L.K., White, P.J., Didelot, X.: A dynamic power-law sexual network model of gonorrhoea outbreaks. PLoS Computational Biology **15** (2019). https://doi.org/10.1371/journal.pcbi.1006748

[5] ONS: Sexual orientation, UK: 2020. https://www.ons.gov.uk/peoplepopulationandcommunity/culturalidentity/sexuality/bulletins/sexualidentityuk/2020. [Online] (Accessed: 28 July 2022) (2022)

[6] Fine, P.E.M., Jezek, Z., Grab, B., Dixon, H.: The transmission potential of monkeypox virus in human populations. International Journal of Epidemiology **17**(3), 643–650 (1988). https://doi.org/10.1093/ije/17.3.643

[7] Jezek, Z., Grab, B., Szczeniowski, M.V., Paluku, K.M., Mutombo, M.: Human monkeypox: secondary attack rates. Bulletin of the World Health Organization **66**(4), 465–470 (1988)

[8] WHO: WHO Director-General declares the ongoing monkeypox outbreak a Public Health Emergency of International Concern. https://www.who.int/europe/news/item/23-07-2022-who-director-general-declares-the-ongoing-monkeypox-outbreak-a-public-health-event-of-international-concern. [Online] (Accessed: 16 September 2022) (2022)

[9] Hubach, R.D., Owens, C.: Findings on the monkeypox exposure mitigation strategies employed by men who have sex with men and transgender women in the United States. Archives of Sexual Behavior, 1–6 (2022)

[10] CDC: Impact of Mpox Outbreak on Select Behaviors. https://www.cdc.gov/poxvirus/monkeypox/response/2022/amis-select-behaviors.html. [Online] (Accessed: 25 November 2022) (2022)

[11] MacGibbon, J., Cornelisse, V.J., Smith, A.K., Broady, T.R., Hammoud, M.A., Bavinton, B.R., Paynter, H., Vaughan, M., Wright, E.J., Holt, M.: Monkeypox knowledge, concern, willingness to change behaviour, and seek vaccination: Results of a national cross-sectional survey. medRxiv (2022)

[12] Smith, L.E., Potts, H.W., Brainard, J.S., May, T., Oliver, I., Amlot, R., Yardley, L., Rubin, G.J.: Mpox knowledge, attitudes, beliefs, and intended behaviour in the general population and men who are gay, bisexual, and who have sex with men. medRxiv (2022)

[13] Czado, C., Gneiting, T., Held, L.: Predictive model assessment for count data. Biometrics **65**(4), 1254–1261 (2009)

[14] Gelman, A., Carlin, J.B., Stern, H.S., Dunson, D.B., Vehtari, A., Rubin, D.B.: Bayesian Data Analysis, Third Edition. Chapman & Hall/CRC Texts in Statistical Science. Taylor & Francis, New York (2013)

[15] Chen, T., Guestrin, C.: XGBoost. In: Proceedings of the 22nd ACM SIGKDD International Conference on Knowledge Discovery and Data Mining - KDD '16, pp. 785–794. ACM Press, New York, USA (2016). https://doi.org/10.1145/2939672.2939785

[16] Friedman, J.H.: Greedy function approximation: A gradient boosting machine. Annals of Statistics **29**(5), 1189–1232 (2001). https://doi.org/10.1214/aos/1013203451

[17] Friedman, J.H., Meulman, J.J.: Multiple additive regression trees with application in epidemiology. Statistics in Medicine **22**(9), 1365–1381 (2003). https://doi.org/10.1002/sim.1501

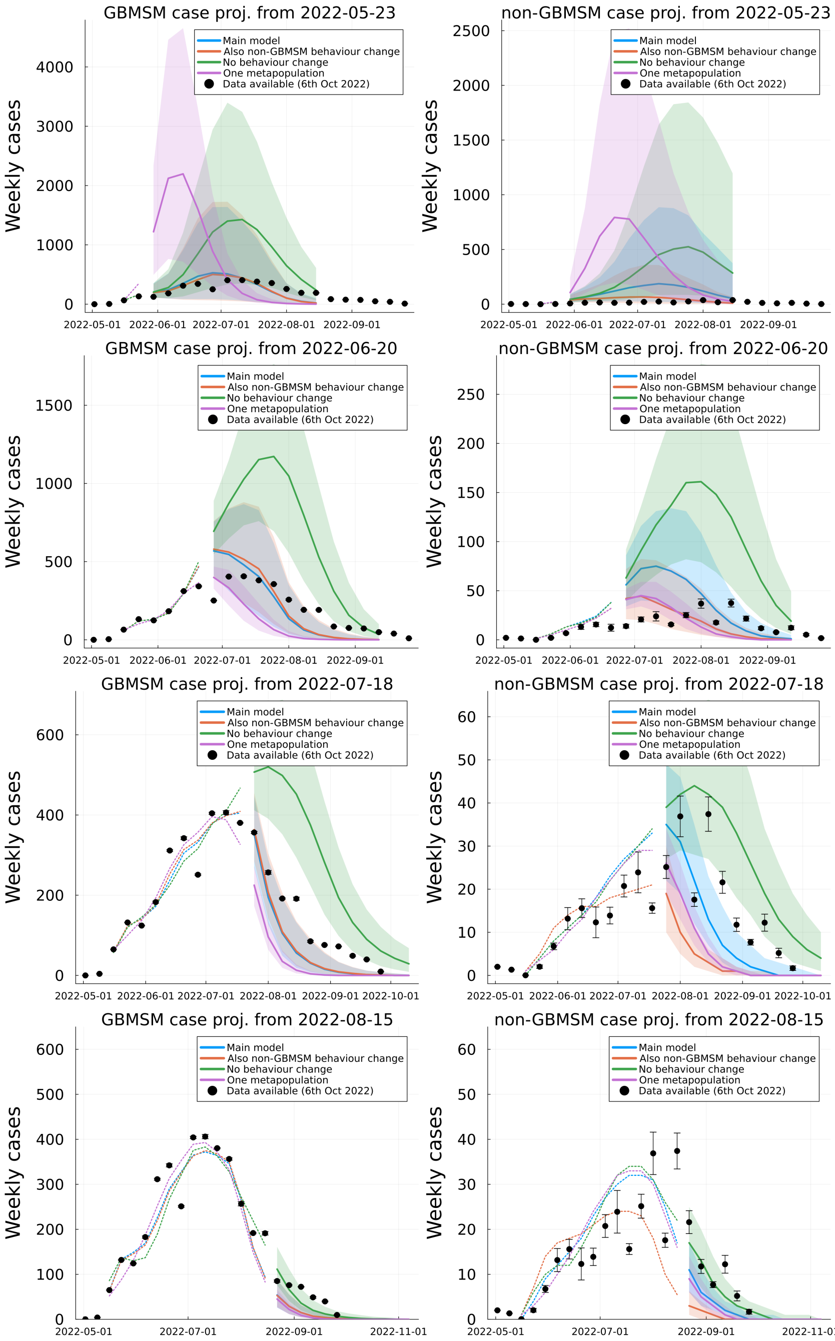

Fig. S5 Sequential projections of weekly case incidence by model choice. Increasing time horizon for including GBMSM (*left*) and non-GBMSM (*right*) case data for parameter inference (*top to bottom*), with subsequent case data used as holdout for scoring models by forecast accuracy. Both posterior median forecasts (*solid curves*) and retrospective posterior median fits (*dashed curves*) are shown by model. Models considered are the main model (*blue*), a full model with behaviour change in all transmission pathways (*red*), "no behaviour change" (*green*), and "one metapopulation" (*purple*). Background shading shows 50% prediction intervals for forecasts, and is not shown for retrospective fits for clarity. Model predictions are based on $n = 2000$ parameter sets drawn from their posterior distribution using data available at the defined time points.

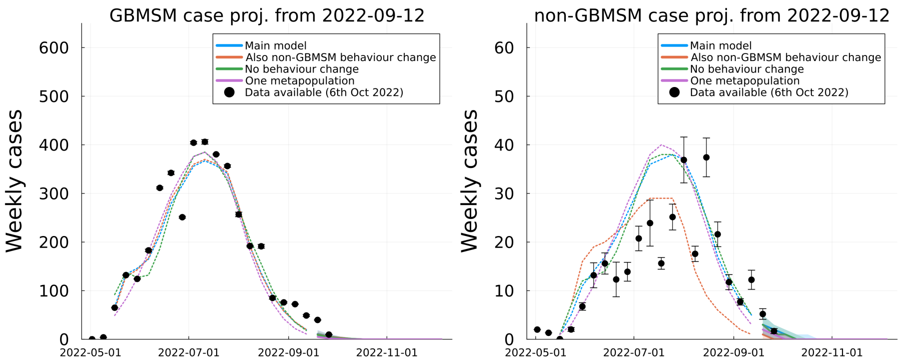

Fig. S6 Full data inference by model choice. Retrospective posterior median fits (*dashed curves*) and posterior forecasts without behaviour reversion (*solid curves*) are shown by model. Models considered are the main model (*blue*), a full model with behaviour change in all transmission pathways (*red*), "no behaviour change" (*green*), and "one metapopulation" (*purple*). Background shading shows 50% prediction intervals for forecasts, and is not shown for retrospective fits for clarity. Model predictions are based on $n = 2000$ parameter sets drawn from their posterior distribution.

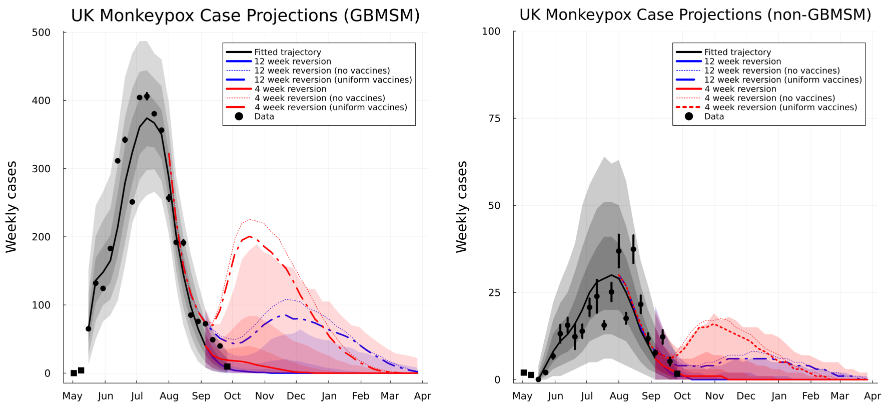

Fig. S7 Counter-factual modelling comparing vaccination with no vaccination and uniform vaccination across GBMSM sexual activity groups. Reversion towards baseline behaviour begins on 1st September and reaches 99% of pre-outbreak behaviour either 4 weeks (*red curves*) or 12 weeks (*black curves*). Weekly Mpox confirmed case data from UKHSA linelist with inferred GBMSM status (black markers; GBMSM *left*, non-GBMSM *right*). Error bars on data points indicate 95% confidence intervals for GBMSM status inference. The posterior median over model projections are shown for 4 weeks reversion and 12 weeks reversion to baseline behaviour (*solid curves*). Also shown is the posterior median model projections for the counterfactual scenario where vaccines are either absent (*dashed curves*) or vaccines are offered uniformly (*dot-dashed curves*). Background shading indicates 50%, 80% and 95% credible or prediction intervals; prediction intervals for the counterfactual results are omitted for clarity. Model predictions are based on $n = 2000$ parameter sets drawn from their posterior distribution.

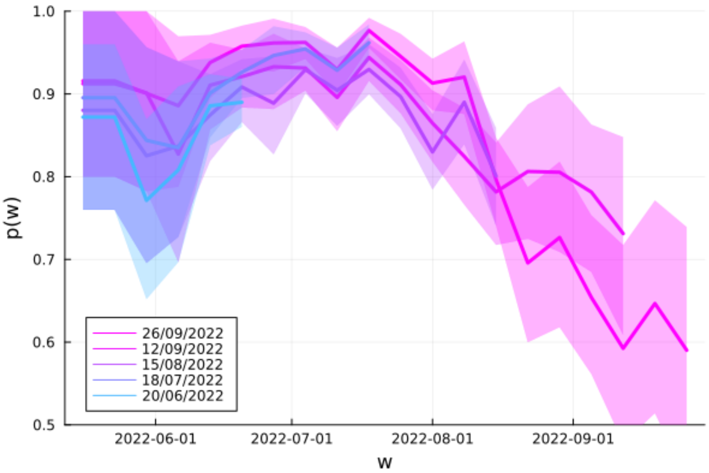

Fig. S8 Fraction of C_{NA} predicted to identify as GBMSM. Colors (cyan to magenta) correspond to values estimated from data obtained until to weeks starting from 20/06/2022, 18/07/2022, 15/08/2022, 12/09/2022, and 26/09/2022 included. Lines show average GBMSM status of each case each week over determined status and GBDT prediction. Shaded areas enclose 95% CIs (confidence intervals). As soon as new cases are included, all past predictions are updated and the CIs tighten, reflecting the increased amount of information introduced with the new cases.
