# Supplementary material for: The role of vaccination and public awareness in forecasts of Mpox incidence in the United Kingdom
Source: Nat Commun. 2023 Jul 11;14:4100. doi: 10.1038/s41467-023-38816-8 (PMC10336136; doi:10.1038/s41467-023-38816-8)
Supplement: Supplementary file 3 — Reporting Summary [file 41467_2023_38816_MOESM3_ESM.pdf]

nature portfolio

Corresponding author(s): Samuel Paul Clark Brand

Last updated by author(s): Apr 24, 2023

Reporting Summary

Nature Portfolio wishes to improve the reproducibility of the work that we publish. This form provides structure for consistency and transparency in reporting. For further information on Nature Portfolio policies, see our Editorial Policies and the Editorial Policy Checklist.

Statistics

For all statistical analyses, confirm that the following items are present in the figure legend, table legend, main text, or Methods section.

n/a	Confirmed	
☒	☐	The exact sample size (*n*) for each experimental group/condition, given as a discrete number and unit of measurement
☒	☐	A statement on whether measurements were taken from distinct samples or whether the same sample was measured repeatedly
☒	☐	The statistical test(s) used AND whether they are one- or two-sided<br>*Only common tests should be described solely by name; describe more complex techniques in the Methods section.*
☒	☐	A description of all covariates tested
☒	☐	A description of any assumptions or corrections, such as tests of normality and adjustment for multiple comparisons
☐	☒	A full description of the statistical parameters including central tendency (e.g. means) or other basic estimates (e.g. regression coefficient) AND variation (e.g. standard deviation) or associated estimates of uncertainty (e.g. confidence intervals)
☒	☐	For null hypothesis testing, the test statistic (e.g. *F*, *t*, *r*) with confidence intervals, effect sizes, degrees of freedom and *P* value noted<br>*Give P values as exact values whenever suitable.*
☐	☒	For Bayesian analysis, information on the choice of priors and Markov chain Monte Carlo settings
☒	☐	For hierarchical and complex designs, identification of the appropriate level for tests and full reporting of outcomes
☒	☐	Estimates of effect sizes (e.g. Cohen's *d*, Pearson's *r*), indicating how they were calculated

Our web collection on statistics for biologists contains articles on many of the points above.

Software and code

Policy information about availability of computer code

Data collection	The raw data was from UKHSA linelist for Monkeypox stored internally on their systems using a data warehouse accessed via SQL.
Data analysis	Data imputation of NAs for GBMSM status: Gradient boosted decision tree implemented using XGBoost 0.83<br>The monkeypox transmission model and parameter inference was designed by the authors and available at https://github.com/SamuelBrand1/MpoxUK .

For manuscripts utilizing custom algorithms or software that are central to the research but not yet described in published literature, software must be made available to editors and reviewers. We strongly encourage code deposition in a community repository (e.g. GitHub). See the Nature Portfolio guidelines for submitting code & software for further information.

Data

Policy information about availability of data

All manuscripts must include a data availability statement. This statement should provide the following information, where applicable:

- Accession codes, unique identifiers, or web links for publicly available datasets
- A description of any restrictions on data availability
- For clinical datasets or third party data, please ensure that the statement adheres to our policy

The full underlying UKHSA linelist data for monkeypox is not openly available due to case sensitivity; application to access this data should be made to the UKHSA. In this study, the full underlying linelist was only used for data imputation of GBMSM status for those cases which did not report. The post-imputation case data,

aggregated by week of case identification and GBMSM status of case, was used to make model-based inference. This anonymous and aggregated dataset is available on the associated code repository https://github.com/SamuelBrand1/MpoxUK .

Human research participants

Policy information about studies involving human research participants and Sex and Gender in Research.

Reporting on sex and gender	NA
Population characteristics	NA
Recruitment	NA
Ethics oversight	NA

Note that full information on the approval of the study protocol must also be provided in the manuscript.

Field-specific reporting

Please select the one below that is the best fit for your research. If you are not sure, read the appropriate sections before making your selection.

☒ Life sciences ☐ Behavioural & social sciences ☐ Ecological, evolutionary & environmental sciences

For a reference copy of the document with all sections, see nature.com/documents/nr-reporting-summary-flat.pdf

Life sciences study design

All studies must disclose on these points even when the disclosure is negative.

Sample size	The underlying data used is the complete information on confirmed monkeypox cases held at UKHSA. Therefore there is not sample size as in a designed study.
Data exclusions	No data were excluded.
Replication	The model uses data from a single epidemic outbreak - so replication is not appropriate.
Randomization	Given this is not a case-control experiment (we are not comparing the outcome of two or more effects) then randomization of the data is not appropriate.
Blinding	Given this is not a case-control experiment then blinding of the data is not appropriate.

Reporting for specific materials, systems and methods

We require information from authors about some types of materials, experimental systems and methods used in many studies. Here, indicate whether each material, system or method listed is relevant to your study. If you are not sure if a list item applies to your research, read the appropriate section before selecting a response.

Materials & experimental systems

n/a	Involved in the study	
☒	☐	Antibodies
☒	☐	Eukaryotic cell lines
☒	☐	Palaeontology and archaeology
☒	☐	Animals and other organisms
☒	☐	Clinical data
☒	☐	Dual use research of concern

Methods

n/a	Involved in the study	
☒	☐	ChIP-seq
☒	☐	Flow cytometry
☒	☐	MRI-based neuroimaging
